# Serum nuclear factor IB as a novel and noninvasive indicator in the diagnosis of secondary hyperparathyroidism

**DOI:** 10.1002/jcla.23787

**Published:** 2021-05-15

**Authors:** Jian’gen Yu, Yu Song, Aihua Yang, Xiaoyun Zhang, Lin Li

**Affiliations:** ^1^ Department of Nephrology The First People’s Hospital of Xiaoshan District Hangzhou China

**Keywords:** BMD scores, chronic renal failure, nuclear factor IB, secondary hyperparathyroidism

## Abstract

**Background:**

Chronic renal failure (CRF) referred to chronic progressive renal parenchymal damage caused by various causes, with metabolite retention and imbalance of water, electrolyte, and acid‐base balance as the main clinical manifestations. Secondary hyperparathyroidism (sHPT) was a common complication in maintenance hemodialysis patients with CRF. Nuclear factor IB (NFIB) was a newly found tumor suppressor gene in various cancers. The present study aimed to illustrate the role of NFIB in sHPT clinical diagnosis and treatment response.

**Methods:**

A retrospective, case‐control study, including 189 patients with sHPT and 106 CRF patients without sHPT, compared with 95 controls. Serum NFIB and 1,25(OH)_2_D_3_ levels were measured by RT‐qPCR and ELISAs, respectively. ROC analysis was conducted to verify the diagnostic value of NFIB in sHPT. Spearman's correlation analysis was conducted to verify the association between NFIB and bone mineral density (BMD) scores. After 6 months of treatment, the variance of NFIB and 1,25(OH)_2_D_3_ in different groups was recorded.

**Results:**

The expression of NFIB was significantly lower in serum samples from sHPT and non‐sHPT CRF patients, compared to controls. Clinicopathological information verified sHPT was associated with NFIB, parathyroid hormone (PTH), serum calcium, serum phosphorus, time of dialysis, and serum 1,25(OH)_2_D_3_ levels. Spearman's correlation analysis illustrated the positive correlation between NFIB levels and BMD scores. At receiver operator characteristic (ROC) curve analysis, the cutoff of 1.6508 for NFIB was able to identify patients with sHPT from healthy controls; meanwhile, NFIB could also discriminate sHPT among CRF patients as well (cutoff = 1.4741). Furthermore, we found that during 6 months of treatment, NFIB levels were gradually increased, while PTH and serum P levels were decreased.

**Conclusions:**

Serum NFIB was a highly accurate tool to identify sHPT from healthy controls and CRF patients. Due to its simplicity, specificity, and sensitivity, this candidate can be proposed as a first‐line examination in the diagnostic workup in sHPT.

## Graphical abstract

The expression of nuclear factor IB (NFIB) was significantly lower in serum samples from secondary hyperparathyroidism (sHPT) and non‐sHPT chronic renal failure (CRF) patients, compared to controls. Clinicopathological information verified sHPT was associated with NFIB, parathyroid hormone (PTH), serum calcium, serum phosphorus, time of dialysis, and serum 1,25(OH)_2_D_3_ levels. Spearman's correlation analysis illustrated the positive correlation between NFIB levels and bone mineral density (BMD) scores. At receiver operator characteristic (ROC) curve analysis, the cutoff of 1.6508 for NFIB was able to identify patients with sHPT from healthy controls; meanwhile, NFIB could also discriminate sHPT among CRF patients as well (cutoff = 1.4741). Furthermore, we found that during 6 months of treatment, NFIB levels were gradually increased, while PTH and serum P levels were decreased.

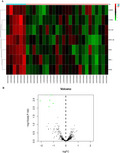

## 1 INTRODUCTION

Chronic renal failure (CRF) was a chronic disease characterized by renal atrophy and loss of renal function.^1^ Statistics showed that the prevalence of CRF was estimated at 8%–16% worldwide,^2^ while the prevalence of chronic renal failure in Japan was as high as 1/50 in males aged above 80, and the number of maintenance hemodialysis patients exceeded 9.2%.^3^ Secondary hyperparathyroidism (sHPT) was one of the most common complications in patients with CRF, due to disorders of calcium and phosphorus metabolism and progressive worsening of hypovitaminosis D, manifested by parathyroid hyperplasia and excessive parathyroid hormone (PTH) synthesis and secretion.^4^, ^5^, ^6^ Clinically, it may hurt bone metabolism, central nervous system, and can cause cardiovascular diseases.^7^, ^8^, ^9^ Some studies have shown that the longer the duration of hemodialysis, the greater the chance of calcium and phosphorus metabolism disorders such as sHPT, which has become a prominent problem affecting the quality of life of maintenance hemodialysis patients.^10^

Secondary hyperparathyroidism (sHPT) was characterized by persistently elevated serum PTH levels and parathyroid hyperplasia.^11^ It was mainly caused by disorders of serum calcium, phosphorus, and vitamin D.^12^ High levels of serum PTH may lead to a high incidence of cardiovascular and bone diseases.^13^ In the state of CRF, low calcium and high phosphorus as well as vitamin D resistance were the main factors triggering sHPT.^11^

Parathyroid hormone (PTH) determined extracellular calcium homeostasis and bone strength, secreted by the parathyroid glands in response to a decrease in serum calcium.^14^ The main target organs for PTH were the skeleton and kidney, which contributed to increased blood calcium levels and decreased blood phosphorus levels.^15^ In patients with CRF, as renal function decreased, renal excretion of phosphorus decreased and patients may exhibit hyperphosphatemia. Meanwhile, increased PTH may damage the vascular endothelium, which increased mineral deposition in the vessel wall and heart valves.^12^ As the glomerular filtration rate irreversibly reduced in CRF patients, along with severe impairment to renal function, excretion of alkaline phosphate and the production of 1,25‐(OH)_2_D_3_ by the kidneys were seriously hindered, resulting in a decrease in the body's ability to absorb calcium, impairment to bone calcium metabolism, and decrease in blood calcium levels.^16^, ^17^ Therefore, the metabolic disorders of blood calcium, phosphorus, and active vitamin D in patients with CRF are closely related to the occurrence of sHPT.^18^

Secondary hyperparathyroidism (sHPT) was an independent risk factor for all‐cause and cardiovascular mortality, and early clinical confirmation had a positive effect on the favorable prognosis of sHPT.^19^ MIBI imaging was the golden criterion for sHPT diagnosis.^20^ For instance, Jiang et al.,^21^ Zeng et al.^22^ and Zhang et al.^23^ demonstrated the high sensitivity and specificity of MIBI in diagnosing sHPT. It may not only detect enlarged parathyroid glands and identify the site of the four parathyroid glands, but also verify ectopic parathyroid glands. However, this invasive diagnostic method was pretty expensive and reproducible. Therefore, there was an urgent need to discover noninvasive, reproducible, and cost‐effective diagnostic methods. In the present study, we displayed differentially expressed genes in samples from sHPT tissues compared with normal parathyroid tissues. Among them, we selected a most significantly differentially expressed gene nuclear factor IB (NFIB) and verified its significance in sHPT.

## 2 MATERIALS AND METHODS

### 2.1 Data collection

Ninety five healthy people who performed routine physical examinations and 295 patients with CRF on maintenance hemodialysis were enrolled at the First People's Hospital of Xiaoshan District from December 2014 to April 2019. Among 295 CFR patients, 189 cases with parathyroid hormone (iPTH) >300 pg/ml were screened as sHPT group, all of whom were not treated with surgical resection. Demographic characteristics, dialysis period, cardiovascular history, diabetes history, laboratory indices such as 1,25(OH)_2_D_3_, PTH, serum calcium, serum phosphorus, and bone mineral density (BMD) scores of each group were recorded and statistically analyzed. Exclusion criteria were as follows: renal transplantation, patients with primary hyperparathyroidism, severe cardiovascular and cerebrovascular complications, malignancies, or severe infections. There were no statistically significant differences between the three groups in terms of gender and age. This study was approved by the Ethics Committee of the First People's Hospital of Xiaoshan District, and all participants signed informed consent forms as request.

Serum samples were collected after 8‐hour fasting (before dialysis in CRF patients), left at room temperature for 2 hours, and then centrifuged at 1000 *g* for 20 minutes. Afterward, the supernatants were separated, and the specimens were stored at −80°C until analysis. BMD scores were measured and evaluated using dual‐energy X‐ray absorptiometry (DXA). Serum calcium, phosphorus, hemoglobin, creatinine, urea nitrogen, and serum parathyroid hormone (PTH) levels were evaluated in the laboratory department in our hospital.

### 2.2 Determination of 1,25(OH)_2_D levels

The concentration of 1,25(OH)_2_D in healthy controls, non‐sHPT CRF, and sHPT patients was examined by commercial kit. As described in the manufacturer's protocol, levels of 1,25(OH)_2_D levels in serum samples were determined by 1,25(OH)_2_D levels reagent kit (Diasorin).

### 2.3 qRT‐PCR assay

To detect whether there were differential expressions of NFIB in serum samples from healthy controls, non‐sHPT CRF cases, and sHPT patients, qRT‐PCR assays were conducted accordingly as per the instructions’ protocol. Briefly, TRIzol reagent was utilized to lyse and extract total RNAs from samples. Then, a NanoDrop microspectrophotometer was used to quantify the RNA concentrations. cDNA was synthesized from RNA using SuperScript IV reagent (Invitrogen; Thermo Fisher Scientific, Inc.), and qRT‐PCR was then run by Universal SYBR Green Master Kit on an ABI 7500 PCR machine (Applied Biosystems). The thermal cycles were as follows: 94°C for 5 minutes, 40 cycles of 94°C for 10 seconds, and 65°C for 30 seconds. Finally, the expressions of NFIB were quantified by equation $2^{‐\Delta \Delta \text{CT}}$ method and normalized to GAPDH.^24^ The primers sequences were as follows: NFIB forward, 5′‐ GGGACTAAGCCCAAGAGACC‐3′, and reverse, 5′‐ GTCCAGTCACAAATCCTCAGC‐3′; GAPDH forward, 5′‐ AGCTTGTCATCAACGGGAAG‐3′, and reverse, 5′‐ TTTGATGTTAGTGGGGTCTCG‐3′.

### 2.4 Calcitriol treatment

According to the patients’ weight, sHPT patients were given calcitriol treatment ad dose 1.5–2.0 µg, orally. Patients weight <60 kg were given 1.5 µg, while patients weighing between 60 and 80 kg were given 2 µg, twice a week, last for 6 months.

### 2.5 Statistical analysis

All statistical analyses were performed using the SPSS version 22.0 (SPSS Inc.) and GraphPad Prism version 6.0. All variables were presented as mean ± SD, and categorical variables were presented as number and proportion. Differences between groups were compared using ANOVA followed by Tukey's post hoc test and a chi‐squared for categorical variables. The analysis results of differentially expressed genes were presented by heatmap and volcano map drawn in RStudio software (version: 1.2.1335). A *p* < 0.05 was considered statistically significant. Receiver operating characteristic (ROC) curves were utilized to determine the cutoff value for the prediction of sHPT.

## 3 RESULTS

### 3.1 Differentially expressed genes in sHPT samples

**FIGURE 1:**
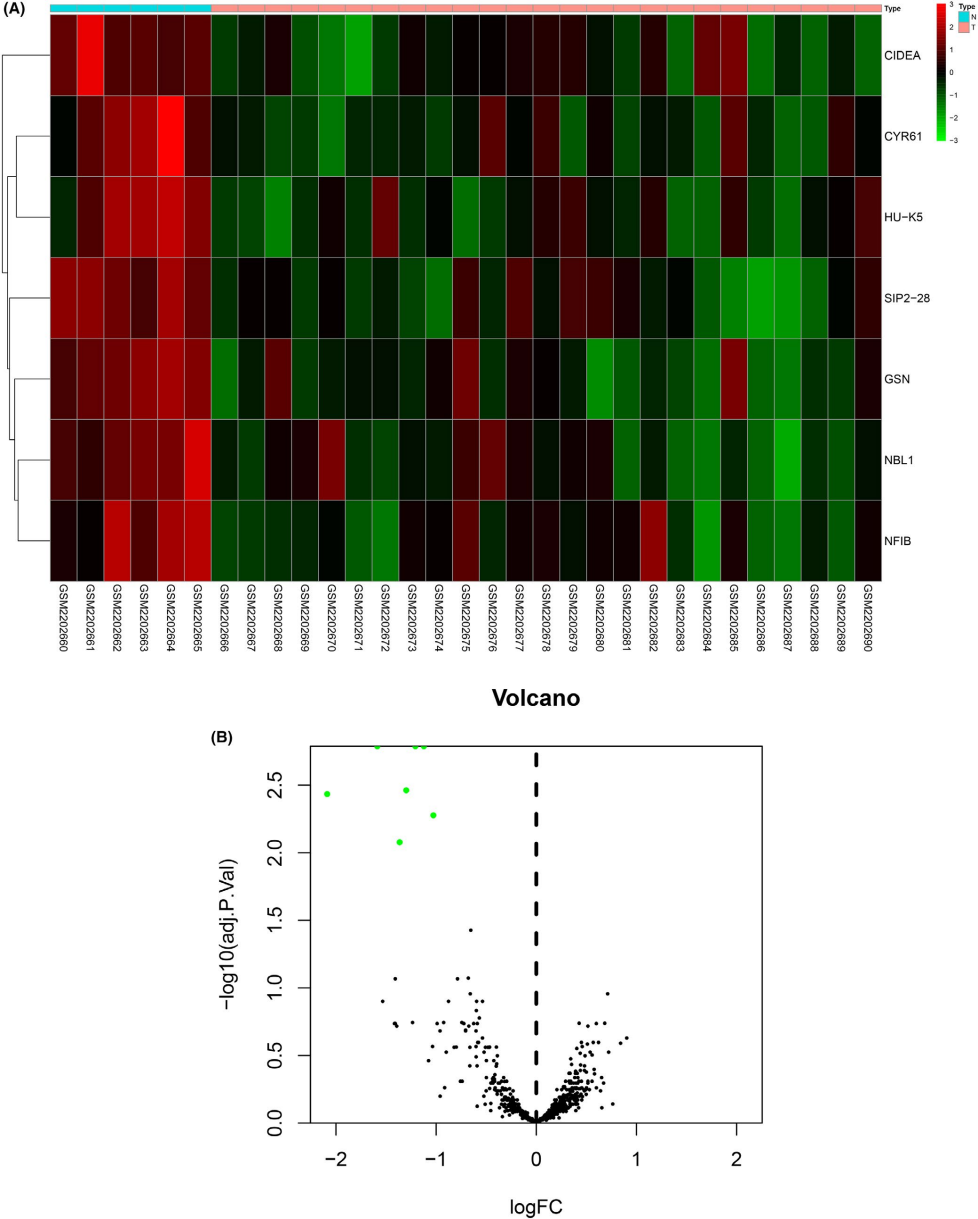
Differentially expressed genes in sHPT. (A) Heatmap for differentially expressed genes in sHPT samples. (B) Volcano plot of differentially expressed genes in sHPT samples

After analyzing 31 GSM (samples) data, we found seven differentially expressed genes (CIDEA, CYR61, HU‐K5, SIP2‐28, GSN, NBL1, and NFIB) in sHPT (Figure 1A and B). All of them were significantly downregulated in sHPT compared with normal samples (Table 1).

**TABLE 1:** Differentially expressed genes in sHPT tissues

Gene ID	logFC	AveExp	*t*	*p* Value	Adj *p* Val	B
CYR61	−1.5874	0.1257	−5.4507	3.73×10^−6^	0.0016	4.3317
SIP2‐28	−1.1228	0.3359	−5.2870	6.18×10^−6^	0.0016	3.8654
GSN	−1.2074	0.8244	−5.2719	6.47×10^−6^	0.0016	3.8224
HU‐K5	−1.2985	0.1738	−4.9337	1.83×10^−5^	0.0034	2.8631
CIDEA	−2.0882	0.2351	−4.8394	2.44×10^−5^	0.0037	2.5968
NBL1	−1.0278	1.0178	−4.6604	4.20×10^−5^	0.0053	2.0942
NFIB	−1.3644	0.8943	−4.4570	7.75×10^−5^	0.0084	1.5279

Abbreviations: Adj *p* Val, Adjust *p* value; AveExp, Average expression.

### 3.2 Serum NFIB levels were dramatically decreased in sHPT patients

**FIGURE 2:**
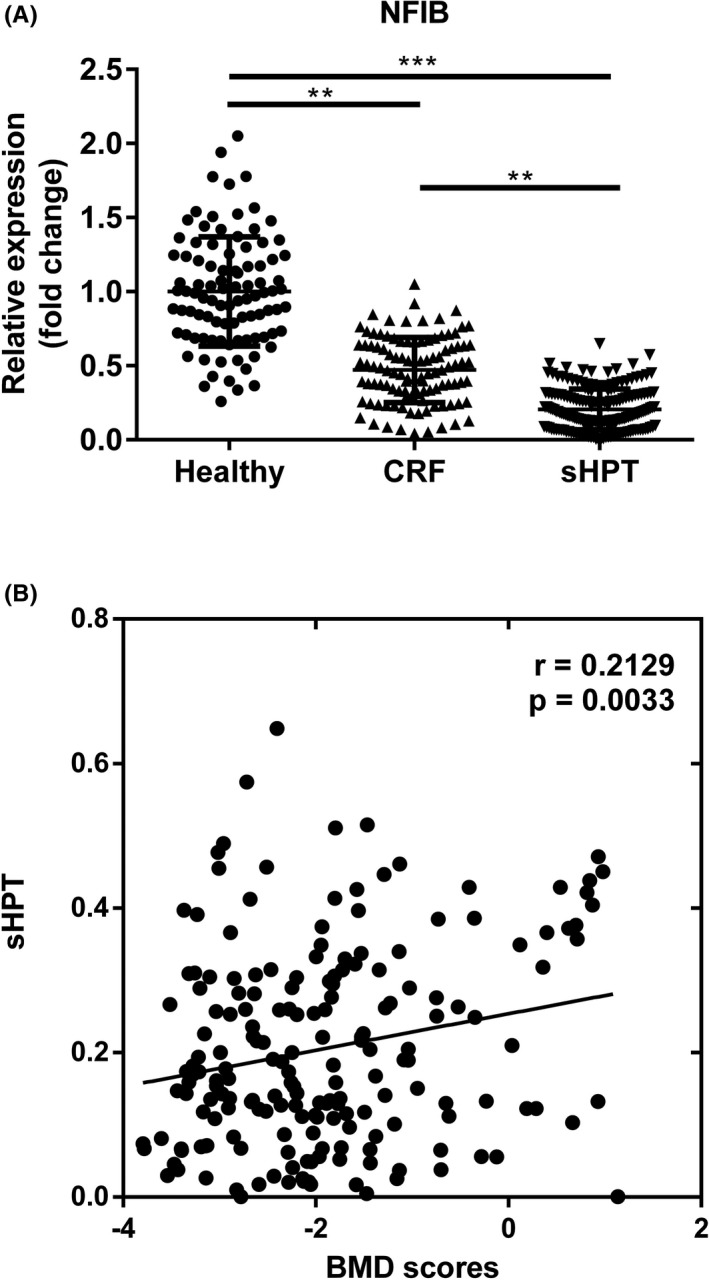
Different expressions of NFIB in sHPT. (A) Aberrant expressions of NIFB in sHPT cases, CRF patients, and healthy controls. (B) Correlation between NFIB level and BMD scores in sHPT patients. ^**^ *p *< 0.01, sHPT vs CRF; CRF vs healthy. ^***^ *p *< 0.001, sHPT vs healthy. BMD, bone mineral density; CRF, chronic renal failure; NFIB, nuclear factor IB; sHPT, secondary hyperparathyroidism

As to determine the aberrant expressions of NFIB in sHPT patients, CRF cases, and healthy controls, qRT‐PCR assays were conducted accordingly. As shown in Figure 2A, the expression of NFIB was significantly decreased in sHPT (^***^ *p *< 0.001) or CRF (^**^ *p *< 0.01) groups compared with healthy controls; moreover, the reduction was more obvious in sHPT group compared with the CRF group (^**^ *p *< 0.01).

### 3.3 Correlation between NFIB levels and BMD scores in sHPT patients

Measurement of BMD score was an adjuvant diagnostic method for monitoring sHPT disease. Hence, we detected the correlation between NFIB levels and BMD scores in sHPT group. As shown in Figure 2B, NFIB expression was positively correlated with BMD scores (*r* = 0.2129, *p* = 0.0033), further suggesting the potential of NFIB in sHPT monitoring.

### 3.4 sHPT was strongly associated with NFIB level, serum PTH level, serum calcium serum phosphorus, time of dialysis, and serum 1,25(OH)_2_D_3_ levels

After recording and analyzing the demographic and clinical parameters in three groups, we found that sHPT may be related to aberrant NFIB, PTH, serum calcium, serum phosphorus, time of dialysis, and serum 1,25(OH)_2_D_3_ levels (Table 2). However, there were no significant differences in age, gender, cardiovascular history, and diabetes history among the three groups.

**TABLE 2:** Demographic and clinical information of all subjects

Characteristics	Healthy (*n* = 95)	CRF (*n* = 106)	sHPT (*n* = 189)	*p* Value
Gender				0.2650
Male	61	64	103	
Female	34	42	86	
Age (years)	58.9 ± 5.2	60.8 ± 4.9	59.6 ± 8.5	0.1410
Dialysis time (years)	/	3.01 ± 2.8	6.87 ± 3.6	<0.0001
Serum calcium (mg/dl)	9.2 ± 2.4	8.3 ± 3.5	6.6 ± 4.7	<0.0001
Serum phosphorus (mg/dl)	3.9 ± 1.1	5.8 ± 0.7	6.1 ± 0.8	<0.0001
Serum PTH (pg/ml)	81.5 ± 11.3	131.5 ± 12.8	169.5 ± 10.7	<0.0001
Serum 1,25(OH)_2_D_3_ (ng/ml)	87.6 ± 3.8	43.8 ± 6.9	36.2 ± 5.0	<0.0001
Cardiovascular history	6	8	11	0.8439
Diabetes history	10	9	13	0.5676
NFIB level (fold)	1.0020 ± 0.3686	0.4713 ± 0.2198	0.2056 ± 0.1370	<0.0001

### 3.5 The potentials of NFIB in sHPT clinical diagnosis

**FIGURE 3:**
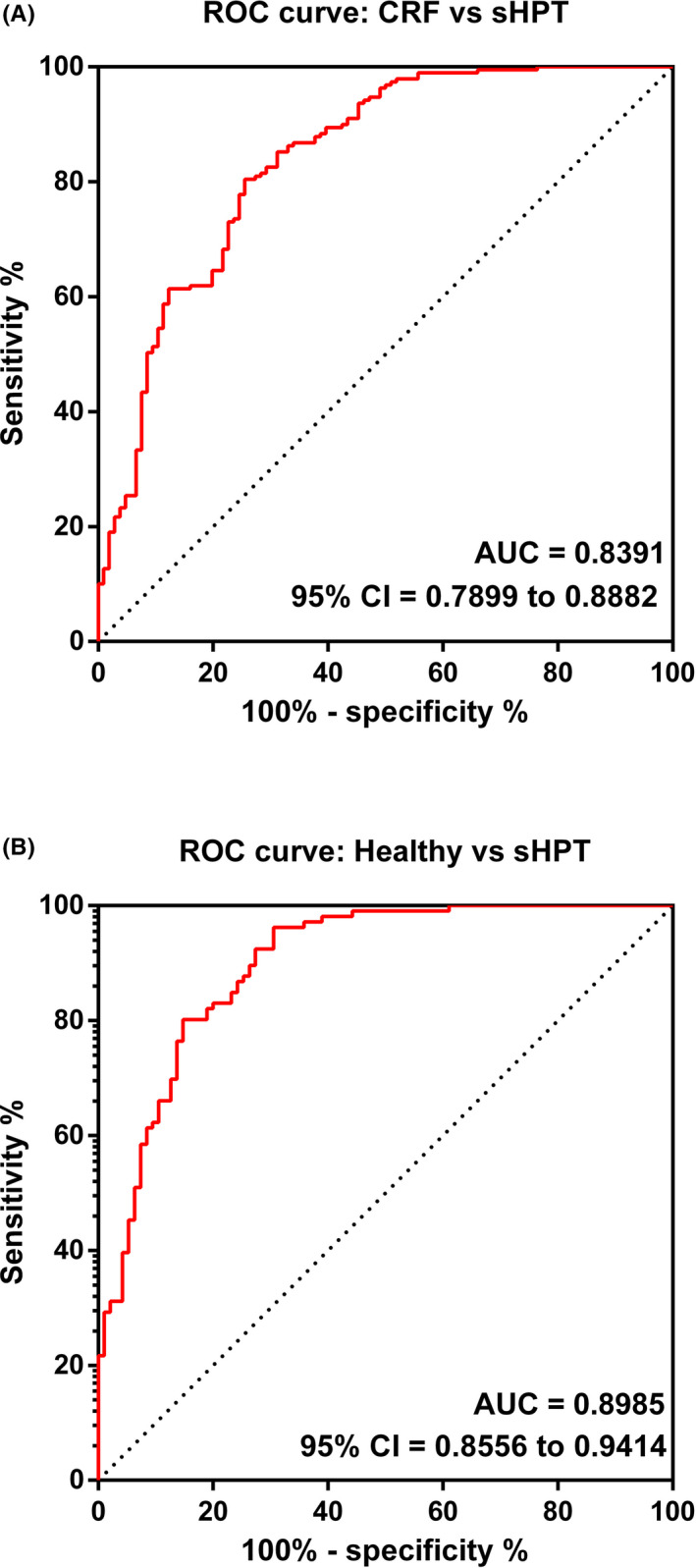
AUCs of NFIB in sHPT. (A) ROC analysis of NFIB in differentiating sHPT from CRF patients. (B) ROC analysis of NFIB in decimating sHPT from healthy volunteers. AUC, area under the curve; CRF, chronic renal failure; NFIB, nuclear factor IB; ROC, receiver operating characteristic; sHPT, secondary hyperparathyroidism

In order to confirm the diagnostic values of NFIB in sHPT, ROC analysis was utilized accordingly. As displayed in Figure 3A, NFIB index could discriminate sHPT patients from CRF cases with the area under the curve of 0.8391 (95% CI = 0.7879–0.8882, specificity = 88.68%, sensitivity = 58.73%, cutoff value = 1.4741). Meanwhile, Figure 3B further verifies the potential of NFIB in identifying sHPT from healthy controls with the cutoff value of 1.6508, the specificity of 72.63%, and the sensitivity of 92.45% (AUC = 0.8985, 95% CI = 0.8556–0.9414).

### 3.6 The variance of NFIB and 1,25(OH)_2_D_3_ levels in sHPT patients after six months of treatment

**FIGURE 4:**
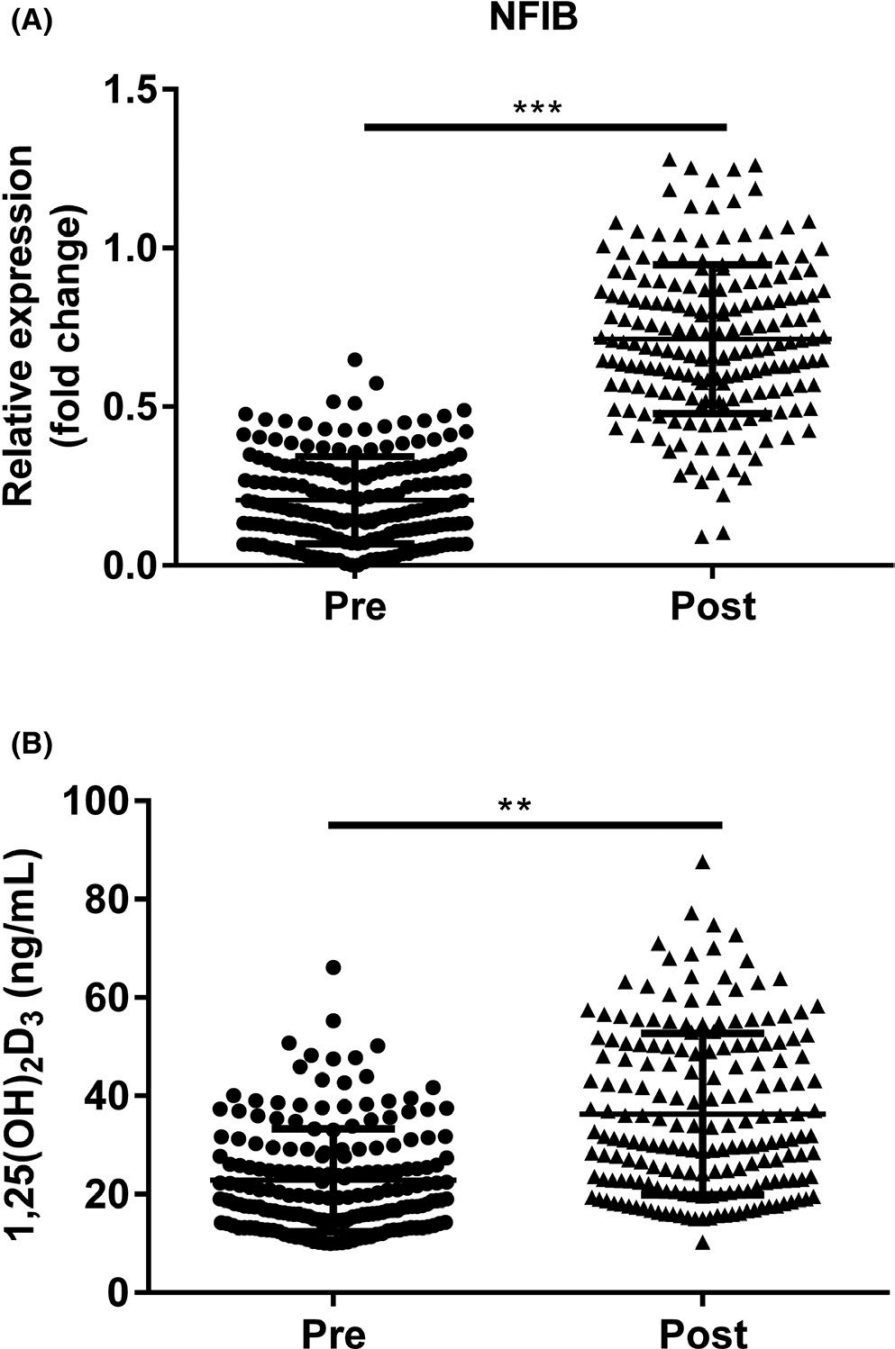
The variance of NFIB and 1,25(OH)_2_D_3_ levels in sHPT after treatment. (A) Expressions of serum NFIB in sHPT group before and after 6 months of treatment. (B) Different 1,25(OH)_2_D_3_ levels in sHPT patients at admission and after treatment. ^***^ *p* < 0.001, ^**^ *p* < 0.01, post vs pre. NFIB, nuclear factor IB and sHPT, secondary hyperparathyroidism

To confirm the significance of NFIB in sHPT treatment response, we continuously monitored the variance of NFIB and 1,25(OH)_2_D_3_ levels in sHPT patients during 6 months of treatment. The results in Figure 4A and B demonstrated that NFIB and 1,25(OH)_2_D_3_ levels were prominently elevated after treatment compared with at admission (^**^ *p *< 0.01 and ^***^ *p *< 0.001).

## 4 DISCUSSION

In patients with CRF, as serum calcium rose, it suppressed the secretion of PTH in parathyroid tissues, ultimately leading to akinetic bone disease, which then slowed down the bone formation and reduced the absorption of excess serum calcium and phosphorus.^11^, ^12^, ^13^ In order to maintain normal bone metabolism and transformation, patients with CRF required higher levels of PTH; hence, serum PTH levels in CRF patients were usually maintained at two to three times of normal value.^25^ sHPT was triggered by hypocalcemia, hyperphosphatemia, and bone resistance to PTH in patients with CRF failure.^10^ With the development of dialysis technology, the life of CRF patients has been extended. However, the incidence of CRF combined with sHPT was increasing, manifested as elevated PTH levels, hyperplasia, and hypertrophy of parathyroid glands. sHPT not only caused serious damage to bones but also aggravated abnormal calcium and phosphorus metabolism, resulting in cardiovascular disease, anemia, and damage to the central nervous system,^7^, ^8^, ^9^ which constituted a vicious circle with CRF. The KDIGO guidelines recommend that all patients with CRF should be screened for sHPT disease.^26^ sHPT management was a stepwise technique designed to optimize serum calcium and phosphorus concentrations, combining a low phosphorus diet with pharmacotherapy.

Recently, a variety of genes were found to be dysregulated in sHPT. For instance, Týcová et al. analyzed 485 differentially expressed genes between nodular and diffuse parathyroid hyperplasia using enrichment analysis.^27^ Santamaría et al. verified 16 upregulated and 132 downregulated genes in the nodules in sHPT through DNA arrays.^28^ In this study, through bioinformatic analysis in 31 GSE samples, we found seven aberrantly expressed genes in sHPT; among them, the NFIB level was significantly decreased in sHPT tissues compared with normal parathyroid tissues. Nuclear factor I (NFI) was a class of transcription factors that were widely present in mammals and were mainly characterized by having a highly conserved N‐terminal DNA binding region.^29^, ^30^ Studies have shown that the NFI family can be involved in the regulation of DNA replication and gene expression, and promote cell proliferation and differentiation during embryonic development.^31^, ^32^ In addition, NFIs were aberrantly expressed in a variety of tumors.^29^, ^33^ NFIB was a member of the NFI family and can be present as a marker in a variety of tumors and diseases. For instance, Wu et al. reported that NFIB was amplified in small cell lung cancer, functioning as an oncogenetic candidate driver in small cell lung cancer.^34^ Liu et al. confirmed the tumor‐promotive role of NFIB in triple‐negative breast cancer by targeting p21 transcription.^35^ Another report by Wu et al. demonstrated that NFIB was elevated in gastric cancer tissues, promoting cell growth, aggressiveness, and metastasis in gastric cancer.^36^ A new study reported by Niu et al. illustrated that NFIB was significantly repressed in renal cell carcinoma, regulated by crocin to promote cell proliferation and migration.^37^ Moreover, we examined the serum levels of NFIB in healthy subjects and maintenance hemodialysis patients with CRF. The results demonstrated that serum levels of NFIB were significantly lower in patients with CRF compared with healthy controls; among patients with CRF, serum levels of NFIB were lower in patients with concomitant sHPT. Meanwhile, the occurrence of CRF combined with sHPT was associated with dialysis period, blood phosphorus, 1,25(OH)_2_D_3_, NFIB, and PHT levels.

Patients with CRF may have different bone loss due to different renal functions, which could gradually develop into osteoporosis (OP) in dialysis patients.^38^ As renal function decreased, PTH levels gradually increased and affected the breakdown of cortical bone and synthesis of cancellous bone, which in turn led to decreased bone transit and the development of irregular bone.^39^ The detection of BMD can reflect changes in bone strength.^40^ DXA was one of the methods used to measure BMD, which quantified bone mass and bone density.^41^ From the results, we found that in sHPT patients, NFIB levels were positively correlated with BMD scores, suggesting the potential of NFIB in predicting sHPT from healthy controls. Meanwhile, ROC analysis further verified the diagnostic significance of NFIB in discriminating sHPT from CRF patients as well.

Recently, many studies have reported a significant increase in the rate of decline of intact parathyroid hormone (iPTH) in patients with sHPT after treatment with calcitriol.^42^, ^43^, ^44^ In the present study, after 6 months of calcitriol treatment, a significantly increased NFIB and 1,25(OH)_2_D_3_ levels were detected in sHPT patients compared with pre‐treatment conditions. Hence, we proposed that serum NFIB levels may reflect calcitriol treatment, contributing to clinical treatment and outcomes.

The main limitations of the study were as follows: First, we did not perform the histopathological examination on all patients or extract parathyroid tissue samples from sHPT patients. Also, the malignancy of sHPT was not divided into more subgroups according to the serum PHT levels in the patients. Finally, all patients were enrolled from the First People's Hospital of Xiaoshan District. Considering these factors, in the future, we need to expand the size of sample collection and improve the statistical methods to make our statistics more generalized.

In sum, NFIB functioned as a feasible biomarker in CRF combined with sHPT, shedding new sights on therapeutic methods.

## CONFLICT OF INTEREST

None.

## Data Availability

The datasets used and/or analyzed during the current study are available from the corresponding author on reasonable request.
